# A review of emerging health threats from zoonotic New World mammarenaviruses

**DOI:** 10.1186/s12866-024-03257-w

**Published:** 2024-04-04

**Authors:** Arianna Lendino, Adrian A. Castellanos, David M. Pigott, Barbara A. Han

**Affiliations:** 1 The George Washington University, Milken Institute for Public Health, Washington, DC 20052 USA; 2 Cary Institute of Ecosystem Studies, Millbrook, NY 12545 USA; 3 Institute for Health Metrics and Evaluation, University of Washington, 2301 5th Ave, Suite 600, Seattle, WA 98121 USA; 4 Department of Health Metrics Sciences, School of Medicine, University of Washington, Seattle, WA 98121 USA

**Keywords:** New World mammarenaviruses, Zoonosis, Rodent, Surveillance, One health, Outbreaks, Socioeconomic, Exposure, Transmission risk, Public health

## Abstract

Despite repeated spillover transmission and their potential to cause significant morbidity and mortality in human hosts, the New World mammarenaviruses remain largely understudied. These viruses are endemic to South America, with animal reservoir hosts covering large geographic areas and whose transmission ecology and spillover potential are driven in part by land use change and agriculture that put humans in regular contact with zoonotic hosts.

We compiled published studies about Guanarito virus, Junin virus, Machupo virus, Chapare virus, Sabia virus, and Lymphocytic Choriomeningitis virus to review the state of knowledge about the viral hemorrhagic fevers caused by New World mammarenaviruses. We summarize what is known about rodent reservoirs, the conditions of spillover transmission for each of these pathogens, and the characteristics of human populations at greatest risk for hemorrhagic fever diseases. We also review the implications of repeated outbreaks and biosecurity concerns where these diseases are endemic, and steps that countries can take to strengthen surveillance and increase capacity of local healthcare systems. While there are unique risks posed by each of these six viruses, their ecological and epidemiological similarities suggest common steps to mitigate spillover transmission and better contain future outbreaks.

**Supplementary Information:**

The online version contains supplementary material available at 10.1186/s12866-024-03257-w.

## Introduction

Rodents are the most speciose group of mammals, with a global distribution spanning a wide array of ecological niches [1]. Rodents are therefore a perennial consideration for zoonotic disease risk because of the pervasive synanthropy in this group, with many species appearing to thrive in cities, rural villages, agricultural areas, as well as in sylvatic habitats. Anthropogenic changes in these environments, including increasing urbanization, agriculture, and related changes in land use, increase the frequency of contact between rodents and human populations leading to more opportunities for spillover transmission of familiar and novel zoonotic pathogens [2–4]. This risk is exemplified by the mammarenaviruses (*Arenaviridae* family), which naturally persist in wildlife hosts and cause numerous hemorrhagic fever diseases globally. Mammarenaviruses are categorized into two major groups based on geography and phylogenetic relationships [5]. The New World mammarenaviruses cause wild rodent-borne hemorrhagic fevers throughout South America and have been reported in the region since 1950 [6, 7]. This group includes Junin virus (JUNV), Chapare virus (CHPV), Machupo virus (MACV), Sabia virus (SABV), Guanarito virus (GTOV), and Tacaribe virus (TCRV). The Old World mammarenaviruses are found in Africa and include Lassa virus and Lujo virus, which are distributed across southern Africa [8]. Although classified as an Old World mammarenavirus, Lymphocytic Choriomeningitis virus (LCMV) is found globally, making it a health concern in every continent except Antarctica [9].

Specific rodent hosts are thought to perpetuate the transmission of these mammarenaviruses by shedding virus through urine, feces, and saliva [10]. Typically, humans become infected either by inhalation of this excreta or secreta, or via direct contact with infected rodents [11]. New World mammarenaviruses are capable of causing severe disease in humans, with fatality rates as high as 30% seen in Chapare virus infections [12]. Patients initially present with flu-like symptoms, such as fever, nausea, vomiting, and diarrhea within 6–12 days [13]. Nearly 25–30% of patients develop severe hemorrhagic symptoms and neurological disorders [7], with symptoms ranging from tongue tremors in mild cases, to mental confusion, seizures, and coma in severe cases. In fatal cases, patients exhibit terminal shock syndrome [14]. Non-fatal outcomes depend on early diagnosis and treatment, which is mainly supportive [15]. Other methods of treatment, such as antivirals, have mainly been assessed in animal models [16], but reports on the efficacy of intravenous plasma and ribavirin exist for Argentine hemorrhagic fever and Bolivian hemorrhagic fever, respectively [16–18]. Preventative treatment is restricted to one available vaccine for Junin virus [19].

Agriculture workers are at the highest risk for exposure and transmission of New World mammarenaviruses [20]. As with many rodent-borne pathogens, ecological, socioeconomic, and occupation factors are all interrelated [21], as the distribution of rodents, corn, and rice crops follow in tandem [20]. In fact, many of the rodents of the subfamily Sigmodontinae that are speciose in South America are often intensely associated with agroecosystems and the stable food sources found there [22, 23]. Widespread human-to-human community transmission has not been reported [24]; however, nosocomial transmission resulting in fatal cases has been documented, thus suggesting a risk to healthcare workers through person-to-person transmission via direct contact with infectious blood and bodily fluids [25]. Their transmissibility and high mortality rates classify viral hemorrhagic fevers of the *Arenaviridae* family as category A pathogens, requiring biosafety level (BSL) 4 precautions [26]. The absence of documented fatal human infections associated with Tacaribe virus (TCRV) permits its handling at reduced biosafety levels. This characteristic renders TCRV a valuable comparative model, both molecularly and serologically, for the study of the broader group of New World mammarenaviruses [27]. Initially isolated from dead *Artibeus* bats and mosquitoes in Trinidad, and subsequently from *Amblyomma americanum* ticks in Florida [28], TCRV is distinguished by its isolation from a diverse range of vertebrate and invertebrate species, notably without any identified rodent host to date [27].

Despite substantial documented fatality rates and the passage of several decades since their identification, New World mammarenaviruses remain relatively understudied compared to other rodent viruses, and compared to the Old World mammarenaviruses (e.g., Lassa fever virus). Yet, they continue to spill over and cause disease annually, often in lower and middle-income countries. Considering the high genetic diversity within numerous New World mammarenaviruses [5, 29], the high biodiversity of potential rodent reservoir species [30], and the tendency to neglect these and similar tropical diseases in research and public health discourse, it is likely that New World mammarenaviruses represent an underappreciated public health challenge. Here, we review the New World mammarenaviruses. We emphasize common research gaps among these viruses including identification of their wildlife reservoirs, their current and future risk to humans, and implications for public health (see Supplementary File 1). Our review extends beyond heuristic analysis to identify particular activities that will enhance epidemiological intelligence and public health preparedness, and delineating areas where foundational knowledge about these pathogens remains elusive.

## Endemicity and reservoirs

### Guanarito virus (GTOV)

**Fig. 1:**
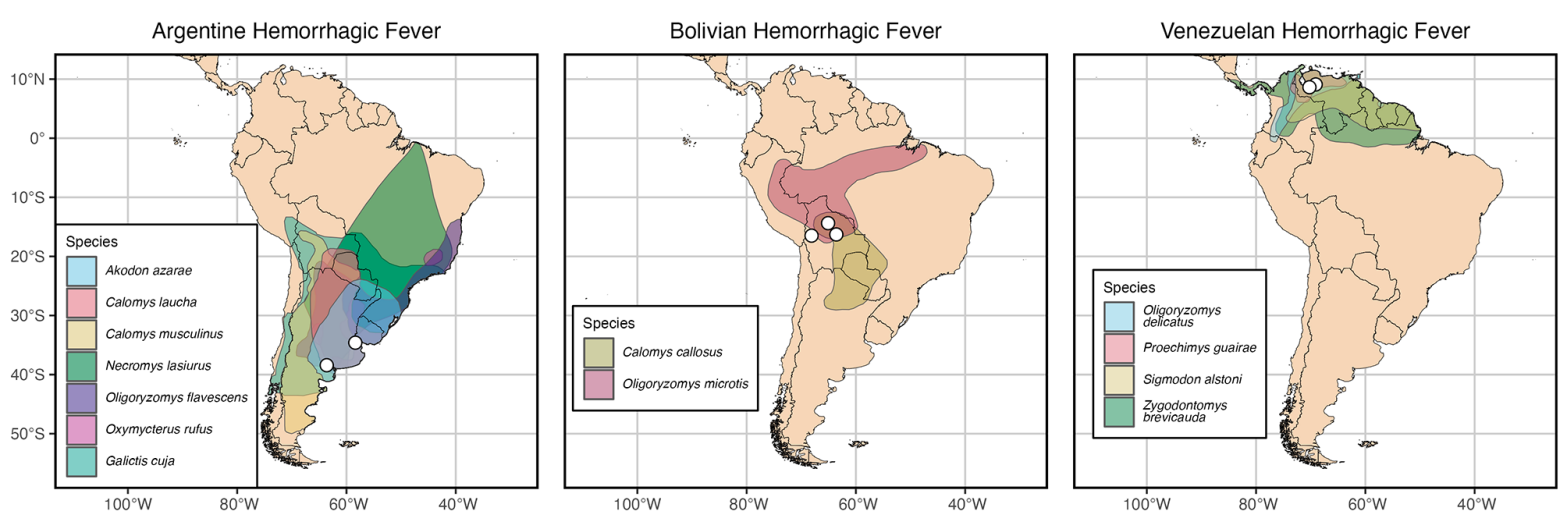
Maps of South America showing the ranges of mammal species associated with New World mammarenaviruses. These species have either been identified as potential hosts from serosurveillance, or have been confirmed as hosts through virus isolation. Outbreaks for each of these hemorrhagic fevers are depicted as white filled circles. Mammal range distributions are from Marsh et al. [91] with a color palette by CARTOcolors from the rcartocolor package in R [92]. Individual maps for each species range can be found in Supplementary File 2

Infection with Guanarito virus causes Venezuelan hemorrhagic fever (VHF), which is found in western Venezuela [31]. The first cases were reported in 1989 during an outbreak of hemorrhagic fever in Guanarito in the state of Portuguesa [32]. Seropositivity and virus isolation from VHF’s primary rodent reservoir host, *Zygodontomys brevicauda* (Short-tailed Cane Mouse), points to an endemic area of 9,000 km^2^ located in the southern and southwestern areas of Venezuela (Fig. 1; [33]). Both GTOV in rodents and human cases of VHF have been reported in the states of Portuguesa and Barinas [33]. Isolates of GTOV have also been found in *Oligoryzomys delicatus* (Delicate Pygmy Rice Rat) and *Sigmodon alstoni* (Groove-toothed Cotton Rat) within these states [34].

Given *Z. brevicauda’s* limited range, GTOV infection is thought to be limited to the rural plains area of western Venezuela [33]. Between 1989 and 2006, there have been 618 reported cases of VHF in Portuguesa, with a reported case-fatality rate of 23.1%. Owing to a lack of surveillance and epidemiological studies in Venezuela, case numbers of VHF were not reported between 2006 and 2021 [33]. However, in 2021, in Barinas and Potuguesa, 36 cases of VHF were confirmed out of 118 suspected cases [24]. Agricultural workers (especially males) are at the highest risk given their occupational proximity to *Z. brevicauda* in rural areas, with the highest number of cases occurring during the height of the agricultural season between November and January [33]. Cases were also reported in seasonal workers from Colombia visiting the endemic region, pointing to exposure to endemic areas as an important risk factor for GTOV [33]. While transmissibility is highest between infected rodents to humans, one probable secondary case of VHF has been reported, indicating the possibility of human-to-human transmission [33].

### Junin virus (JUNV)

Junin virus is the causal agent of Argentine hemorrhagic fever (AHF), which was first isolated in the 1950s among agricultural workers and those raising cattle in the Pampa region of Argentina [35]. Cases of AHF in agricultural areas tend to increase in late autumn in tandem with the corn harvest [36]. The main natural reservoir, *Calomys musculinus* (Drylands Vesper Mouse) is synanthropic, commonly found in areas in close proximity to farm workers and other at-risk populations [37]. Male farm workers between the ages of 20 and 50 consistently show the highest seroprevalence of exposure to JUNV. While infection is 90% more common in rural areas [35], JUNV has also been found in cities such as Santa Fe and Cordoba [36]. In a study by Vitullo et al. [37], *Calomys musculinus* individuals infected with JUNV at birth exhibited increased mortality and reduced fertility in adulthood. Conversely, when *C. musculinus* are infected with JUNV during adulthood, they maintain a continuous infection and virus shedding without exhibiting alterations in reproductive behavior or survival. Notably, their offspring are also infected. Therefore, it appears that both horizontal (between individuals) and vertical (from parent to offspring) transmission pathways play significant roles in maintaining persistent infections in wild reservoir populations [37]. Another member of this genus, *Calomys laucha* (Small Vesper Mouse), is an additional confirmed reservoir for JUNV, and seropositivity has been found in *Akodon azarae* (Azara’s Grass Mouse), *Necromys lasiurus* (Hairy-tailed Akodont), and *Galictis cuja* (Lesser Grison) (Fig. 1; [35, 38, 39]). The geographic range of *C. musculinus* suggests that the endemic area for this virus covers ∼ 150,000 km^2^, putting nearly 5,000,000 individuals at potential risk in the endemic region [35], although this historical assessment doesn’t take into account an increased range caused by *C. laucha* as a reservoir. We note that this and other such estimates of at-risk populations assume equivalent disease risk across the entire range where the reservoir is present, which is an oversimplification [40] as many other factors are known to restrict pathogen distributions. Within this endemic area, there is co-circulation of multiple mammarenaviruses with JUNV, LCMV, and non-zoonotic Latino virus found in rodents captured in the Río Cuarto department [41].

Changes in crop operations, increased rodent populations, and greater exposure of humans to rodents have all contributed to disease incidence [35]. Between 1958 and 1987, there were 21,000 cases among male workers in rural areas, with a mean annual increase of 360 cases per year between 1983 and 1987 [35]. Government authorities have not released consistent AHF case counts in recent years, although suspected incidence appears to be low. There were only 13 reported cases in 2018 and 2 cases in 2022 [35]. The drop in reported cases over time could be explained by vaccination against JUNV; the vaccine (Candid 1) is a live attenuated vaccine developed via an Argentina-United States partnership in the 1980s [35]. Candid 1 efficacy is 95.5% [42]. Originally developed to protect agricultural workers, the Junin virus vaccine has significantly reduced the incidence of Argentine Hemorrhagic Fever since its introduction in the 1980s [43]. Argentina’s vaccination policy for JUNV was established in 1991; given that this was an orphan drug with finite quantities, the vaccine campaign was restricted to geographic locations and human populations likely to have the highest incidence of disease [42]. It is unclear whether JUNV spillover infections will rebound given that vaccinations are no longer being developed or administered. Using monoclonal antibodies potentially offers an additional treatment approach to Junin virus [16].

### Sabia virus (SABV)

Sabia virus (*Brazilian mammarenavirus*) is the causal agent of Brazilian hemorrhagic fever [16]. SABV was first reported with two human cases in São Paulo (Cotia in 1990 and Espirito Santo de Pinhal in 1999) [7]. Both cases involved farm workers working in rural areas, and both cases were fatal [7]. While attempting characterization of SABV, a laboratory technician was infected in 1992, most likely through aerosols. Similarly, in 1994, a researcher was accidentally infected with SABV through aerosol exposure stemming from a broken centrifuge, pointing to another occupational risk associated with New World mammarenaviruses. Both cases were non-fatal, as the individuals knew to seek immediate medical attention [44]. Until 2019, these were the only four known cases of SABV [7]. Recently, a hiker and a farm worker independently presented with symptoms similar to yellow fever. They had both been in São Paulo, an area that recently experienced a yellow fever outbreak [7]. Metagenomic assays confirmed the presence of *Brazilian mammarenavirus* in both patients following their deaths [7]. The reservoir for SABV is unknown, but it is suspected to be a rodent species [45].

### Machupo virus (MACV)

Machupo virus is one of two recognized etiologic agents of Bolivian hemorrhagic fever [46]. MACV was first discovered in 1963 in San Joaquin in the Beni department of Bolivia; 637 confirmed cases of Bolivian hemorrhagic fever occurred with a 25–30% mortality rate between 1963 and 1964. Disease incidence of Bolivian hemorrhagic fever is unknown due to weak epidemiologic surveillance infrastructure in Bolivia [46]. The reservoir for MACV is *Calomys callosus* [46], and the virus circulates in these rodent populations via horizontal, vertical, and sexual transmission [6]. Risk for Bolivian hemorrhagic fever is particularly high for farmers, especially during rainy agricultural harvest seasons, for males, given their likelihood of working in agriculture, researchers who may potentially be exposed to aerosols in the lab, and healthcare workers who are likely to be in close contact with infected patients [46].

### Chapare virus (CHPV)

Chapare virus is the other recognized etiologic agent of Bolivian hemorrhagic fever. It was discovered via RT-PCR (real-time polymerase chain reaction) between 2003 and 2004 in Cochabamba, Bolivia, during a hemorrhagic fever outbreak [46]. Co-circulation of MACV and CHPV may be possible, as their probable rodent reservoirs have overlapping distributions (Fig. 1). CHPV has also been detected in Beni, where MACV was first discovered, and may serve as an additional cause of hemorrhagic fever there [46]. In 2019, viral hemorrhagic fever was reported in Caranavi and the etiologic agent was later confirmed as CHPV marking these as the first cases in the 16 years after the virus was first identified [47]. Phylogenetic analysis determined that the cases in this outbreak of Bolivian hemorrhagic fever came from multiple strains and likely represented multiple spillover events in North La Paz associated with agriculture [47]. During this outbreak, *Oligoryzomys microtis* (Small-eared Pygmy Rice Rat) was identified as the probable reservoir responsible for zoonotic spillover transmission. Both nosocomial transmission and human-to-human transmission outside of the hospital setting was also confirmed [47]. Eight confirmed cases and one probable case of Bolivian hemorrhagic fever caused by CHPV were reported in this outbreak, and four of those cases were fatal. The initial diagnosis for the primary patient was dengue fever, thus delaying appropriate supportive care and infection control strategies for Bolivian hemorrhagic fever, which are vital for patient outcomes [47].

### Lymphocytic choriomeningitis virus (LCMV)

LCMV is classified as an Old World mammarenavirus but is found throughout the Americas, Europe, Australia, and Japan [48]. The primary rodent reservoir is the house mouse, *Mus musculus*, which is a ubiquitous species. An estimated 5% of house mice throughout the United States are thought to be infected. Infected house mice are capable of transmitting LCMV throughout their lives without overt signs of illness [48]. *M. musculus* was recently identified in the Caribbean island of Barbados with serological evidence of an mammarenavirus infection that is suspected to be LCMV given its widespread distribution [49]. Infection to humans occurs through bites [48] and inhalation of excreta and saliva [9]. The majority of LCMV infections in humans are asymptomatic or cause mild fever; however, initial mild presentation of LCMV infection can be followed by a second phase marked by neurological disease, and meningitis, encephalitis, or meningoencephalitis [48]. Compared to the New World mammarenaviruses, LCMV mortality is relatively low (less than 1%) [48]. Vertical transmission has been observed in pregnant women who may transmit the infection in utero leading to congenital malformations. LCMV infection has been implicated in several fatal results in recipients of organ transplants [50–52].

### Contextual factors

These viruses pose significant mitigation challenges that warrant consideration of several contextual factors that are present throughout the endemic areas in varying degrees.

#### Public Health Infrastructure & Healthcare Capacity

The COVID-19 pandemic accentuated numerous challenges confronting public health infrastructure across the endemic region [53]. These include limited financial investment in public health, a decline in healthcare resources—especially acute in rural areas—and fragmented health systems. Such fragmentation results in disease mitigation strategies being determined at varying state and local levels, impeding a unified and coordinated public health response to the pandemic [53]. Health inequities also persist throughout South America, even in Brazil, which has a universal right to health for all citizens as a constitutional mandate [54]. Inequalities in access, adherence to, and quality of care are consistent with socioeconomic differences, with wealthier individuals possessing greater access to quality care and increased agency over their healthcare decisions than individuals of lower socioeconomic status [54]. Rural populations, particularly indigenous populations, share a disproportionate amount of the disease burden throughout South America due to a lack of healthcare workers, poor epidemiological disease surveillance, and low health literacy [55]. These issues of healthcare inequity and faltering infrastructure hamper preparedness for spillovers of New World mammarenaviruses.

The situation in Venezuela highlights a common set of challenges to disease surveillance, prevention, and mitigation. Despite some recent reports of 36 confirmed cases of VHF, regular data updates on VHF stopped in 2006 [33]. The country has also been in political and economic crisis since 2014, causing the country’s health system to collapse [56], which presents a situation of uncertainty with respect to incidence of diseases such as VHF, which become further neglected compared to diseases such as measles, diphtheria, and tuberculosis that have resurfaced at unsustainable rates [57], and reemergence of vector-borne diseases such as malaria [58]. The societal and political unrest, external economic sanctions, and migration are not unique to Venezuela. Other countries experience similar barriers to disease surveillance and epidemiological studies [59, 60], against a backdrop of weakened public health infrastructure [61].

As was the case globally, COVID-19 justifiably demanded the full attention of surveillance and mitigation efforts in South America and further strained the public healthcare system responsible for caring for South America’s poorest citizens [62]. Surveillance of other diseases, including those caused by New World mammarenaviruses, became a lower priority for Venezuela and other South American countries [63]. The government in Argentina has not reported case counts of Argentine hemorrhagic fever since the onset of the COVID-19 pandemic, except for two cases of AHF reported in July 2022 [35]. The last cases of Bolivian hemorrhagic fever, caused by the Machupo virus, were reported in 2008, and the last cases of Sabia hemorrhagic fever were reported in Brazil in 2017 [15]. Underreporting due to lack of surveillance may be one reason for a paucity of data throughout the endemic region; however, New World mammarenaviruses coexist with other hemorrhagic fevers and undersurveilled pathogens that are likely to be contributing to syndromes whose etiologies remain persistent but ill-defined and therefore difficult to treat [64].

#### Diagnostic capacity

Viral culture, immunohistochemistry, or RT-PCR are often used for diagnosis [16]. Historically, there have been no biosafety level 4 (BSL 4) facilities in South America, limiting access to laboratories that can safely test for these pathogens [65]. Specimens were often sent to the Centers for Disease Control and Prevention (CDC) in the United States for testing [16], which significantly slowed investigations into rapidly developing outbreaks, and risked the removal of important disease investigation assets from the outbreak area [66]. In 2026, Brazil is scheduled to open South America’s first BSL 4 facility [65] designed to safely support basic research on New World mammarenaviruses in the endemic region.

#### Human and animal ecology

The relationship between humans and the environment plays a significant role in infectious disease emergence, especially in South America where the diversity of animal reservoirs is substantial [63]. Except for Sabia virus, whose animal hosts are currently unknown, the primary reservoirs for the other zoonotic New World mammarenaviruses in South America are sigmodontine rodents [67]. This group of rodents entered South America from North America as part of the Great American Biotic Interchange and subsequently dispersed across the continent, initially colonizing and diversifying in eastern South America [68, 69]. The Andes mountains subsequently played an essential role in the high diversity in this group, with repeated invasions and vicariant events acting as a “species pump” over different periods, creating new species at a faster rate in the Andean groups of sigmodontine rodents [70]. Their high diversity and the often cryptic nature has led to an ever changing taxonomic understanding of Sigmodontinae, both in their species descriptions (due to molecular tools) and the shuffling of higher order relationships with increasingly large genomic datasets [71, 72]. Given the rapid changes in species descriptions, species identification can be difficult for surveillance, requiring additional resources to precisely identify rodents to the species-level, and complicating our understanding of species ranges to better define the endemic areas for the viruses they harbor [73]. Notably, although New World mammarenaviruses were thought to have codiverged with their hosts, recent phylogenetic analyses suggest that host-virus relationships are instead caused by the tendency of these viruses to switch to new hosts that are geographically overlapping [74]. This implies that the rodent-virus relationships in this group may have been fluid historically, and may be capable of switching in the future.

It is clear that land use changes are the main driver in the emergence of New World mammarenaviruses [36]. The destruction of habitats for agricultural purposes and other development [75] brings humans into closer contact with animals, particularly rodents who exhibit synanthropic behavior and carry more zoonotic diseases than any other mammal group, thus increasing the risk for zoonotic spillover [21]. *Calomys musculinus*, a reservoir of JUNV, shows differing patterns of genetic and population structure between urban and agricultural habitats, with higher winter survival in urban areas possibly driving part of this differentiation [76]. Additionally, more generalist species such as *C. musculinus* may reach greater abundances, benefitting from intense agriculture and displacing or outcompeting specialist species that are not known to be reservoirs of these hemorrhagic fevers [77, 78].

## Conclusions

Our review reveals that there remain many important fundamental unknowns about zoonotic New World mammarenaviruses, but also emphasizes concurrent advantages to investing resources to address the risks of viral hemorrhagic fevers. Expanding our basic understanding of these viruses will reveal how to bolster public health systems that will build the infectious disease intelligence needed to enhance outbreak preparedness for multiple diseases [79, 80]. Mitigating the risks of mammarenavirus outbreaks will serve to enhance the overall infectious disease resilience in these countries. Many of these diseases similarly share sylvatic reservoirs or vectors that support repeated spillover transmission of zoonotic pathogens that are projected to increase in incidence with land use change, a trend that continues unabated and poses ongoing threats [81–83].

Enhanced disease surveillance, informed by a deeper understanding of mammarenavirus seasonality and transmission patterns, is crucial. Rather than placing the onus solely on resource-constrained countries, collective efforts leverage the strengths of international organizations, neighboring countries, and global health networks. Programs like PAHO’s successful campaigns against other infectious diseases serve as a model for how collaboration and resource-sharing can yield substantial public health dividends [63, 84, 85]. Sustained collaborations across disciplines and across countries, like those supported by the NIH NIAID CREID network or virtually organized efforts like Museums and Emerging Pathogens in the Americas (MEPA), have shored up interdisciplinary collaborations to better understand complex interactions between agriculture and land use change, host ecology, and virology, and their change over time [86, 87].

It is evident that while New World mammarenaviruses possess distinct characteristics, their effective mitigation aligns with broader principles applicable to zoonotic pathogens. Crucial to this effort is the adoption of collaborative, multi-sectoral strategies that encompass research, response, and preparedness activities, as these approaches have shown substantial promise in addressing similar health threats [88, 89]. The cornerstone of such strategies lies in nurturing within-country research capabilities and providing consistent, targeted support to local scientists across relevant disciplines. This support is essential to avoid pitfalls of reactive funding reallocation, which can disrupt the continuity of interdisciplinary research and hinder the development of comprehensive mitigation solutions [90]. Addressing these challenges will bridge vital knowledge gaps concerning mammarenaviruses and also fortify long-term research infrastructure in endemic regions. Such fortification is a strategic investment, yielding significant returns in enhancing global health security and equipping us to effectively tackle both current and future zoonotic challenges.

### Electronic supplementary material

Below is the link to the electronic supplementary material.

Supplementary Material 1: A description of the literature review conducted including a list of the keywords used for literature searches

Supplementary Material 2: Maps showing additional versions of Fig. 1 that include the distribution of each species alone and not overlapping

Supplementary Material 3: A spreadsheet including information on mammals associated with the viruses discussed here.

Supplementary Material 4: A spreadsheet that includes identifying information for all articles that were retained at the end of the literature review

## Data Availability

All additional files referenced can be found on FigShare, which includes all papers that were retained as part of our literature search as Supplementary file 4 (10.25390/caryinstitute.c.6963891).

## Abbreviations

AHF: Argentine hemorrhagic fever
BSL: Biosafety level
CDC: Centers for Disease Control and Prevention
CHPV: Chapare virus
CNPEM: Centro Nacional de Pesquisa em Energia e Materiais
CREID: Centers for Research in Emerging Infectious Diseases
GTOV: Guanarito virus
JUNV: Junin virus
LCMV: Lymphocytic Choriomeningitis virus
MACV: Machupo virus
NIH: National Institutes of Health
NIAID: National Institute of Allergy and Infectious Diseases
PAHO: Pan American Health Organization
RT: PCR-real-time polymerase chain reaction
SABV: Sabia virus
TCRV: Tacaribe virus
VHF: Venezuelan hemorrhagic fever

## References

[1] Kay EH, Hoekstra HE, Rodents (2008). Curr Biol.

[2] Mendoza H, Rubio AV, García-Peña GE, Suzán G, Simonetti JA (2019). Does land-use change increase the abundance of zoonotic reservoirs? Rodents say yes. Eur J Wildl Res.

[3] García-Peña GE, Rubio AV, Mendoza H, Fernández M, Milholland MT, Aguirre AA (2021). Land-use change and rodent-borne diseases: hazards on the shared socioeconomic pathways. Philos Trans R Soc Lond B Biol Sci.

[4] Han BA, Schmidt JP, Bowden SE, Drake JM (2015). Rodent reservoirs of future zoonotic diseases. Proc Natl Acad Sci U S A.

[5] Radoshitzky SR, Bào Y, Buchmeier MJ, Charrel RN, Clawson AN, Clegg CS (2015). Past, present, and future of arenavirus taxonomy. Arch Virol.

[6] Banerjee C, Allen LJS, Salazar-Bravo J (2008). Models for an arenavirus infection in a rodent population: consequences of horizontal, vertical and sexual transmission. Math Biosci Eng.

[7] Nastri AC, Duarte-Neto AN, Casadio LVB, de Souza WM, Claro IM, Manuli ER (2022). Understanding Sabiá virus infections (Brazilian mammarenavirus). Travel Med Infect Dis.

[8] Centers for Disease Control and Prevention. Arenaviruses (Arenaviridae). 2021. https://www.cdc.gov/vhf/virus-families/arenaviridae.html. Accessed 28 Nov 2023.

[9] Charrel RN, de Lamballerie X (2010). Zoonotic aspects of arenavirus infections. Vet Microbiol.

[10] Mills JN, Childs JE (1998). Ecologic studies of rodent reservoirs: their relevance for human health. Emerg Infect Dis.

[11] Charrel RN, de Lamballerie X (2003). Arenaviruses other than Lassa virus. Antiviral Res.

[12] Delgado S, Erickson BR, Agudo R, Blair PJ, Vallejo E, Albariño CG (2008). Chapare virus, a newly discovered arenavirus isolated from a fatal hemorrhagic fever case in Bolivia. PLoS Pathog.

[13] Kerber R, Reindl S, Romanowski V, Gómez RM, Ogbaini-Emovon E, Günther S (2015). Research efforts to control highly pathogenic arenaviruses: a summary of the progress and gaps. J Clin Virol.

[14] Marta RF, Montero VS, Molinas FC (1998). Systemic disorders in Argentine haemorrhagic fever. Bull Inst Pasteur.

[15] Belhadi D, El Baied M, Mulier G, Malvy D, Mentré F, Laouénan C (2022). The number of cases, mortality and treatments of viral hemorrhagic fevers: a systematic review. PLoS Negl Trop Dis.

[16] Frank MG, Beitscher A, Webb CM, Raabe V, members of the Medical Countermeasures Working Group of the National Emerging Special Pathogens (2021). Training and Education Center’s (NETEC’s) Special Pathogens Research Network (SPRN). South American hemorrhagic fevers: a summary for clinicians. Int J Infect Dis.

[17] Kilgore PE, Ksiazek TG, Rollin PE, Mills JN, Villagra MR, Montenegro MJ (1997). Treatment of Bolivian hemorrhagic fever with intravenous Ribavirin. Clin Infect Dis.

[18] Maiztegui J, Fernandez N, De Damilano A, Efficacy of immune, plasma in treatment of argentine Hamorrhagic fever and association between treatment and a late neurological syndrome. Lancet. 1979;314:1216–7. 10.1016/s0140-6736(79)92335-3 92624

[19] Maiztegui JI, McKee KT, Oro B, Harrison JG, Gibbs LH, Feuillade PH (1998). Protective efficacy of a live attenuated vaccine against Argentine hemorrhagic fever. AHF Study Group. J Infect Dis.

[20] Escalera-Antezana JP, Rodriguez-Villena OJ, Arancibia-Alba AW, Alvarado-Arnez LE, Bonilla-Aldana DK, Rodríguez-Morales AJ (2020). Clinical features of fatal cases of Chapare virus hemorrhagic fever originating from rural La Paz, Bolivia, 2019: a cluster analysis. Travel Med Infect Dis.

[21] Ecke F, Han BA, Hörnfeldt B, Khalil H, Magnusson M, Singh NJ (2022). Population fluctuations and synanthropy explain transmission risk in rodent-borne zoonoses. Nat Commun.

[22] Revollo-Cadima SG, Rico CA, Pacheco LF, Salazar-Bravo J (2020). Community structure and abundance of small rodents at the wave front of agroforestry and forest in Alto Beni, Bolivia. Ecología en Bolivia.

[23] Ellis BA, Mills JN, Glass GE, McKee KT, Enria DA, Childs JE (1998). Dietary habits of the common rodents in an Agroecosystem in Argentina. J Mammal.

[24] Rodríguez-Morales AJ, Bonilla-Aldana DK, Risquez A, Paniz-Mondolfi A, Suárez JA (2021). Should we be concerned about Venezuelan hemorrhagic fever? - a reflection on its current situation in Venezuela and potential impact in Latin America amid the migration crisis. New Microbes New Infect.

[25] Borio L, Inglesby T, Peters CJ, Schmaljohn AL, Hughes JM, Jahrling PB (2002). Hemorrhagic fever viruses as biological weapons: medical and public health management. JAMA.

[26] Michalski A, Knap J, Bielawska-Drózd A, Bartoszcze M (2022). Lessons learned from 2001–2021 - from the bioterrorism to the pandemic era. Ann Agric Environ Med.

[27] Holzerland J, Leske A, Fénéant L, Garcin D, Kolakofsky D, Groseth A (2020). Complete genome sequence of Tacaribe virus. Arch Virol.

[28] Sayler KA, Barbet AF, Chamberlain C, Clapp WL, Alleman R, Loeb JC (2014). Isolation of Tacaribe virus, a caribbean arenavirus, from host-seeking Amblyomma americanum ticks in Florida. PLoS ONE.

[29] Emonet SF, de la Torre JC, Domingo E, Sevilla N (2009). Arenavirus genetic diversity and its biological implications. Infect Genet Evol.

[30] Maestri R, Patterson BD (2016). Patterns of species richness and turnover for the South American Rodent Fauna. PLoS ONE.

[31] Tesh RB (1994). The emerging epidemiology of Venezuelan hemorrhagic fever and Oropouche fever in tropical South America. Ann N Y Acad Sci.

[32] Tesh RB, Wilson ML, Salas R, De Manzione NM, Tovar D, Ksiazek TG (1993). Field studies on the epidemiology of Venezuelan hemorrhagic fever: implication of the cotton rat Sigmodon alstoni as the probable rodent reservoir. Am J Trop Med Hyg.

[33] Silva-Ramos CR, Montoya-Ruíz C, Faccini-Martínez ÁA, Rodas JD (2022). An updated review and current challenges of Guanarito virus infection, Venezuelan hemorrhagic fever. Arch Virol.

[34] Weaver SC, Salas RA, de Manzione N, Fulhorst CF, Duno G, Utrera A (2000). Guanarito virus (Arenaviridae) isolates from endemic and outlying localities in Venezuela: sequence comparisons among and within strains isolated from Venezuelan hemorrhagic fever patients and rodents. Virology.

[35] Kumar S, Yadav D, Singh D, Shakya K, Rathi B (2023). Poonam. Recent developments on Junin virus, a causative agent for Argentine haemorrhagic fever. Rev Med Virol.

[36] Mills JN, Ellis BA, McKee KT, Calderon GE, Maiztegui JI, Nelson GO (1992). A longitudinal study of Junin virus activity in the rodent reservoir of Argentine hemorrhagic fever. Am J Trop Med Hyg.

[37] Vitullo AD, Hodara VL, Merani MS (1987). Effect of persistent infection with Junin virus on growth and reproduction of its natural reservoir, Calomys musculinus. Am J Trop Med Hyg.

[38] Parodi AS, Dela Barrera JM, Rugiero HR, Greenway DJ, Yerga M, Mettler N (1959). Los reservorios del virus de la fiebre hemorrágica epidémica de la Provincia de Buenos Aires. Prensa Med Argent.

[39] Mills JN, Ellis BA, Childs JE, McKee KT, Maiztegui JI, Peters CJ (1994). Prevalence of infection with Junin virus in rodent populations in the epidemic area of Argentine hemorrhagic fever. Am J Trop Med Hyg.

[40] Harris NC, Dunn RR (2010). Using host associations to predict spatial patterns in the species richness of the parasites of north American carnivores. Ecol Lett.

[41] Calderón GE, Provensal MC, Martin ML, Brito Hoyos DM, García JB, Gonzalez-Ittig RE (2022). Cocirculación De virus Junin Y otros mammarenavirus en área geográfica sin casos confirmados de fiebre Hemorrágica Argentina. Medicina.

[42] Enria DA, Briggiler AM, Feuillade MR (1998). An overview of the epidemiological, ecological and preventive hallmarks of Argentine haemorrhagic fever (Junin virus). Bull Inst Pasteur.

[43] Gowen BB, Hickerson BT, York J, Westover JB, Sefing EJ, Bailey KW (2021). Second-generation live-attenuated Candid#1 vaccine Virus resists reversion and protects against Lethal Junín Virus infection in Guinea Pigs. J Virol.

[44] Ellwanger JH, Chies JAB (2017). Keeping track of hidden dangers - the short history of the Sabiá virus. Rev Soc Bras Med Trop.

[45] de Mello Malta F, Amgarten D, Nastri AC, de Ho SS, Boas Casadio Y-L, Basqueira LV (2020). Sabiá Virus-Like Mammarenavirus in patient with Fatal Hemorrhagic Fever, Brazil, 2020. Emerg Infect Dis.

[46] Silva-Ramos CR, Faccini-Martínez ÁA, Calixto O-J, Hidalgo M (2021). Bolivian hemorrhagic fever: a narrative review. Travel Med Infect Dis.

[47] Loayza Mafayle R, Morales-Betoulle ME, Romero C, Cossaboom CM, Whitmer S, Alvarez Aguilera CE (2022). Chapare Hemorrhagic Fever and Virus detection in rodents in Bolivia in 2019. N Engl J Med.

[48] Centers for Disease Control and Prevention. Lymphocytic Choriomeningitis (LCM). 2014. https://www.cdc.gov/vhf/lcm/index.html. Accessed 28 Nov 2023.

[49] Douglas KO, Cayol C, Forbes KM, Samuels TA, Vapalahti O, Sironen T et al. Serological evidence of multiple zoonotic viral infections among wild rodents in Barbados. Pathogens. 2021;10. 10.3390/pathogens10060663 PMC8229225 34071689

[50] Fischer SA, Graham MB, Kuehnert MJ, Kotton CN, Srinivasan A, Marty FM (2006). Transmission of lymphocytic choriomeningitis virus by organ transplantation. N Engl J Med.

[51] Amman BR, Pavlin BI, Albariño CG, Comer JA, Erickson BR, Oliver JB (2007). Pet rodents and fatal lymphocytic choriomeningitis in transplant patients. Emerg Infect Dis.

[52] Palacios G, Druce J, Du L, Tran T, Birch C, Briese T (2008). A new arenavirus in a cluster of fatal transplant-associated diseases. N Engl J Med.

[53] Garcia PJ, Alarcón A, Bayer A, Buss P, Guerra G, Ribeiro H (2020). COVID-19 response in Latin America. Am J Trop Med Hyg.

[54] Villar Uribe M, Escobar M-L, Ruano AL, Iunes RF (2021). Realizing the right to health in Latin America, equitably. Int J Equity Health.

[55] Martins F, Lima A, Diep L, Cezarino L, Liboni L, Tostes R (2023). COVID-19, SDGs and public health systems: linkages in Brazil. Health Policy Open.

[56] Edwards B. Failing hospitals and Healthcare Systems. The Dark side of Healthcare. WORLD SCIENTIFIC; 2020. pp. 1–25.

[57] Claborn DM. A narrative review of the role of Economic Crisis on Health and Healthcare Infrastructure in three Disparate National environments. Int J Environ Res Public Health. 2020;17. 10.3390/ijerph17041252 PMC7068242 32075237

[58] Grillet ME, Hernández-Villena JV, Llewellyn MS, Paniz-Mondolfi AE, Tami A, Vincenti-Gonzalez MF (2019). Venezuela’s humanitarian crisis, resurgence of vector-borne diseases, and implications for spillover in the region. Lancet Infect Dis.

[59] Hirschfeld K (2017). Failing States as epidemiologic risk zones: implications for Global Health Security. Health Secur.

[60] Elachola H, Doumbia S, Kattan RF, Abubakar I, Memish ZA (2018). Implications of converging conflicts, emergencies, and mass gatherings for global health security. Lancet Glob Health.

[61] Pinna Pintor M, Suhrcke M, Hamelmann C. The impact of economic sanctions on health and health systems in low-income and middle-income countries: a systematic review and narrative synthesis. BMJ Glob Health. 2023;8. 10.1136/bmjgh-2022-010968 PMC9923316 36759018

[62] Litewka SG, Heitman E (2020). Latin American healthcare systems in times of pandemic. Dev World Bioeth.

[63] Yeh KB, Parekh FK, Borgert B, Olinger GG, Fair JM (2021). Global health security threats and related risks in Latin America. Global Security: Health Sci Policy.

[64] Rodriguez-Morales AJ, Paniz-Mondolfi AE, Faccini-Martínez ÁA, Henao-Martínez AF, Ruiz-Saenz J, Martinez-Gutierrez M (2021). The constant threat of zoonotic and Vector-Borne Emerging Tropical diseases: living on the Edge. Front Trop Dis.

[65] Medina E. Brazil will have the world’s first maximum biosafety containment laboratory complex connected to a synchrotron light source. Centro Nacional de Pesquisa em Energia e Materiais (CNPEM). 2023. https://cnpem.br/en/brasil-tera-nb4-conectado-sincrotron-mundo/. Accessed 28 Nov 2023.

[66] Rao V, Bordelon E (2019). Mobile High-Containment Biological Laboratories Deployment: opportunities and challenges in Expeditionary deployments to Outbreak Response. Appl Biosaf.

[67] Chiappero MB, Piacenza MF, Provensal MC, Calderón GE, Gardenal CN, Polop JJ (2018). Effective Population size differences in Calomys musculinus, the host of Junín Virus: their relationship with the Epidemiological History of Argentine Hemorrhagic Fever. Am J Trop Med Hyg.

[68] Maestri R, Upham NS, Patterson BD (2019). Tracing the diversification history of a Neogene rodent invasion into South America. Ecography.

[69] Percequillo AR, Prado JR do, Abreu EF, Dalapicolla J, Pavan AC, de Almeida Chiquito E et al. Tempo and mode of evolution of oryzomyine rodents (Rodentia, Cricetidae, Sigmodontinae): A phylogenomic approach. Mol Phylogenet Evol. 2021;159:107120. 10.1016/j.ympev.2021.107120 33610650

[70] Vallejos-Garrido P, Pino K, Espinoza-Aravena N, Pari A, Inostroza-Michael O, Toledo-Muñoz M (2023). The importance of the Andes in the evolutionary radiation of Sigmodontinae (Rodentia, Cricetidae), the most diverse group of mammals in the Neotropics. Sci Rep.

[71] D’Elía G, Fabre P-H, Lessa EP (2019). Rodent systematics in an age of discovery: recent advances and prospects. J Mammal.

[72] Parada A, Hanson J, D’Eiía G (2021). Ultraconserved Elements improve the resolution of difficult nodes within the Rapid Radiation of Neotropical Sigmodontine Rodents (Cricetidae: Sigmodontinae). Syst Biol.

[73] Müller L, Gonçalves GL, Cordeiro-Estrela P, Marinho JR, Althoff SL, Testoni AF (2013). DNA barcoding of sigmodontine rodents: identifying wildlife reservoirs of zoonoses. PLoS ONE.

[74] Irwin NR, Bayerlová M, Missa O, Martínková N (2012). Complex patterns of host switching in New World arenaviruses. Mol Ecol.

[75] Tapia-Ramírez G, Lorenzo C, Navarrete D, Carrillo-Reyes A, Retana Ó (2022). Carrasco-Hernández R. A review of mammarenaviruses and Rodent reservoirs in the Americas. EcoHealth.

[76] Chiappero MB, Panzetta-Dutari GM, Gómez D, Castillo E, Polop JJ, Gardenal CN (2011). Contrasting genetic structure of urban and rural populations of the wild rodent Calomys musculinus (Cricetidae, Sigmodontinae). Mamm Biol.

[77] Gomez MD, Coda J, Simone I, Martínez J, Bonatto F, Steinmann AR (2015). Agricultural land-use intensity and its effects on small mammals in the central region of Argentina. Mammal Res.

[78] Han BA, O’Regan SM, Paul Schmidt J, Drake JM (2020). Integrating data mining and transmission theory in the ecology of infectious diseases. Ecol Lett.

[79] Kelly TR, Karesh WB, Johnson CK, Gilardi KVK, Anthony SJ, Goldstein T (2017). One health proof of concept: bringing a transdisciplinary approach to surveillance for zoonotic viruses at the human-wild animal interface. Prev Vet Med.

[80] Lulli LG, Baldassarre A, Mucci N, Arcangeli G, Prevention. Risk exposure, and knowledge of Monkeypox in Occupational settings: a scoping review. Trop Med Infect Dis. 2022;7. 10.3390/tropicalmed7100276 PMC9608671 36288017

[81] Prist PR, Uriarte M, Fernandes K, Metzger JP (2017). Climate change and sugarcane expansion increase Hantavirus infection risk. PLoS Negl Trop Dis.

[82] Blasdell KR, Morand S, Laurance SGW, Doggett SL, Hahs A, Trinh K (2022). Rats and the city: implications of urbanization on zoonotic disease risk in Southeast Asia. Proc Natl Acad Sci U S A.

[83] Pruvot M, Chea S, Hul V, In S, Buor V, Ramassamy J-L (2024). Small mammals at the edge of deforestation in Cambodia: transient community dynamics and potential pathways to pathogen emergence. One Earth.

[84] Andrus JK, Solorzano CC, de Oliveira L, Danovaro-Holliday MC, de Quadros CA (2011). Strengthening surveillance: confronting infectious diseases in developing countries. Vaccine.

[85] Estallo EL, Sippy R, Robert MA, Ayala S, Barboza Pizard CJ, Pérez-Estigarribia PE (2023). Increasing arbovirus risk in Chile and neighboring countries in the Southern Cone of South America. Lancet Reg Health Am.

[86] Colella JP, Bates J, Burneo SF, Camacho MA, Carrion Bonilla C, Constable I (2021). Leveraging natural history biorepositories as a global, decentralized, pathogen surveillance network. PLoS Pathog.

[87] Vasilakis N, Hanley KA. The Coordinating Research on Emerging Arboviral Threats Encompassing the Neotropics (CREATE-NEO). Zoonoses (Burlingt). 2023;3. 10.15212/zoonoses-2022-0047 PMC10586723 37860630

[88] Vora NM, Hannah L, Walzer C, Vale MM, Lieberman S, Emerson A (2023). Interventions to Reduce Risk for Pathogen Spillover and Early Disease Spread to Prevent outbreaks, epidemics, and pandemics. Emerg Infect Dis.

[89] de la Rocque S, Belot G, Errecaborde KMM, Sreedharan R, Skrypnyk A, Schmidt T et al. Operationalisation of consensual one health roadmaps in countries for improved IHR capacities and health security. BMJ Glob Health. 2021;6. 10.1136/bmjgh-2021-005275 PMC8252684 34210688

[90] Diptyanusa A, Zablon KN (2020). Addressing budget reduction and reallocation on health-related resources during COVID-19 pandemic in malaria-endemic countries. Malar J.

[91] Marsh CJ, Sica YV, Burgin CJ, Dorman WA, Anderson RC, Del Toro Mijares I (2022). Expert range maps of global mammal distributions harmonised to three taxonomic authorities. J Biogeogr.

[92] Nowosad J. CARTOColors Palettes. 2018.

