# Supplementary material for: A review of emerging health threats from zoonotic New World mammarenaviruses
Source: BMC Microbiol. 2024 Apr 4;24:115. doi: 10.1186/s12866-024-03257-w (PMC10993514; doi:10.1186/s12866-024-03257-w)

Search details:

We used the PubMed and Web of Science search engines to identify scientific articles published about risks for transmission of mammarenaviruses to humans from animal reservoirs, with no specified date range. To investigate the diversity of New World mammarenaviruses, we used combinations of search terms that included the individual virus (7 total viruses: Sabia virus, Junin virus, Guanarito virus, Machupo virus, Chapare virus, Sabia virus, Lymphocytic Choriomeningitis virus), “reservoirs” and “risk to humans,” and individual hemorrhagic fevers associated with each virus (4 total hemorrhagic fevers: Venezuelan hemorrhagic fever, Argentine hemorrhagic fever, Bolivian hemorrhagic fever, Brazilian hemorrhagic fever) and “reservoirs” and “risk to humans,” for a total of 22 unique search strings. We excluded all articles related to molecular and therapeutic work, included scientific articles of all languages, and translated articles using Google Lens. We conducted a second search with the same search engines to identify articles about health system capacity and public health infrastructure in the endemic area for each of these hemorrhagic fevers, focusing on rural areas.

As the recent COVID-19 pandemic has caused major changes in public health and disease research, we specified a five year timeframe spanning the emergence of the pandemic (2019-2023) to capture research preceding the COVID-19 pandemic and subsequent impacts on public health infrastructure and health system capacity. As above, we created numerous search term combinations including: “Public Health Capacity” and “Rural” and the following countries: (“Brazil”, “Bolivia”, and “Argentina”); “Rural” and “Health system capacity;” “Public health systems” and “Latin America and Rodent Borne” and lastly, “Rural Health” and “Latin America.” Given the humanitarian crisis in Venezuela, we also targeted a specific search to investigate this topic using the terms “Venezuela” and “Public Health Crisis” and “Rural.”

Given their high fatality rate, arenaviruses are recognized as possible agents of bioterrorism (Borio et al., 2002). To gain an understanding of these viruses and their potential use for bioterrorism, we used the following search terms: "Listed New World Arenaviruses," "BSL 4," and "Bioterrorism" and "New World Arenaviruses." For prevention measures, we combined the following search terms: "Preventing Spillover," and "Humans" and "Rodent-borne diseases," adding a broader search timeframe (2013-2023) to identify more articles over ten years. To target surveillance efforts (both reservoir and human) we included the search terms: "Surveillance and rodents," "Surveillance and New World Arenaviruses," and lastly, "Latin America," "Multisectoral surveillance," and "Zoonoses." This second search yielded a total of 13 unique search strings.

This second search only included articles about health system capacity and public health infrastructure in Argentina, Bolivia, Brazil and Venezuela. From 327 unique records, 168 duplicate articles were removed, resulting in 97 unique records ultimately included (Additional Figure 1). Drawing upon the PRISMA framework (Page et al., 2021), Additional Figure 1 summarizes the process of study identification from databases, the number of records that were excluded and the reasons for exclusion, and the total number of studies included in the review.

Additional Figure 1. PRISMA flow chart showing identification of mammarenavirus studies for review.

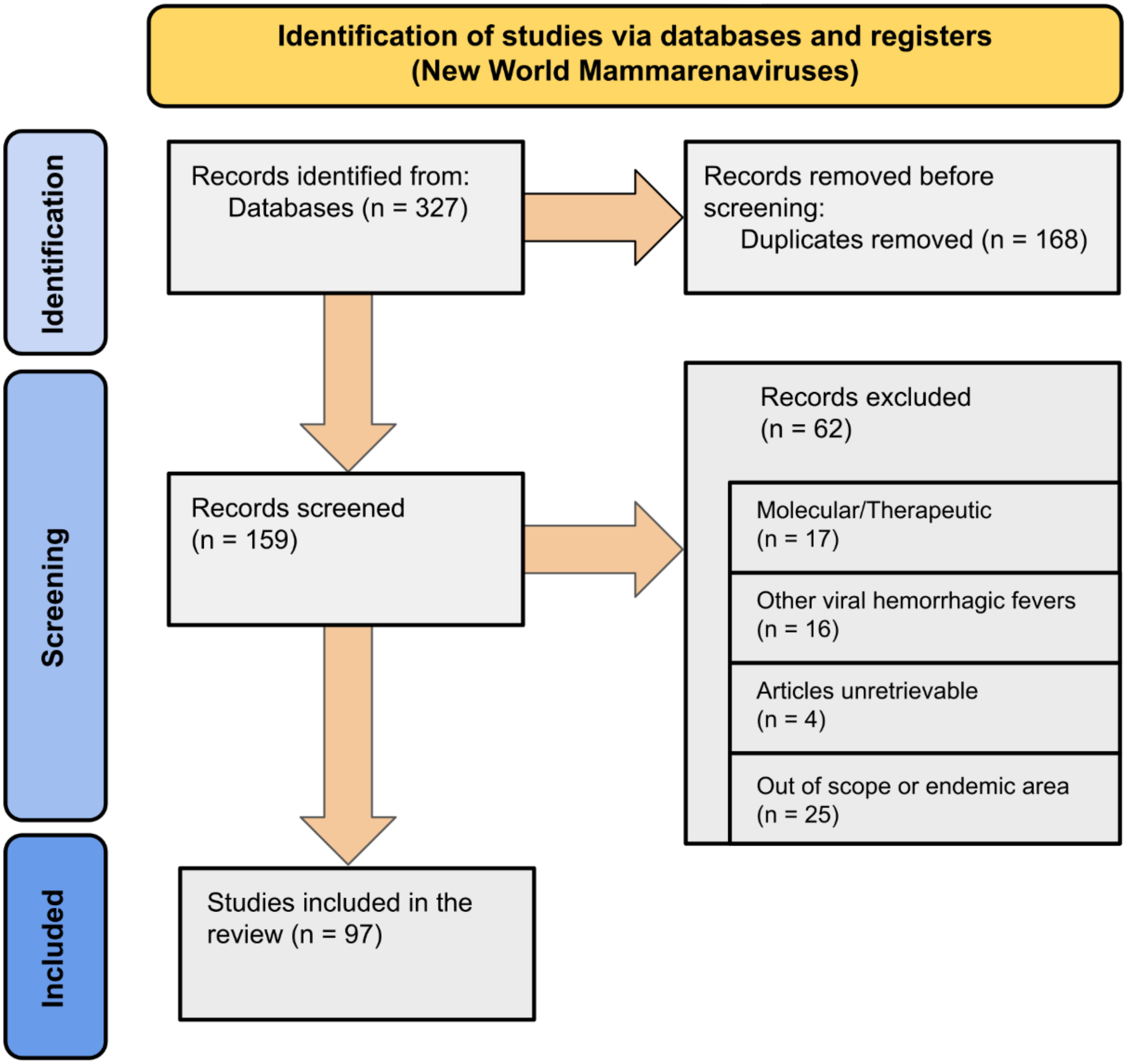

Supplement: Supplementary file 1 — Supplementary Material 1: A description of the literature review conducted including a list of the keywords used for literature searches [file 12866_2024_3257_MOESM1_ESM.pdf]
