# Supplementary figures and images for: A review of emerging health threats from zoonotic New World mammarenaviruses
Source: BMC Microbiol. 2024 Apr 4;24:115. doi: 10.1186/s12866-024-03257-w (PMC10993514; doi:10.1186/s12866-024-03257-w)

## Argentine Hemorrhagic Fever

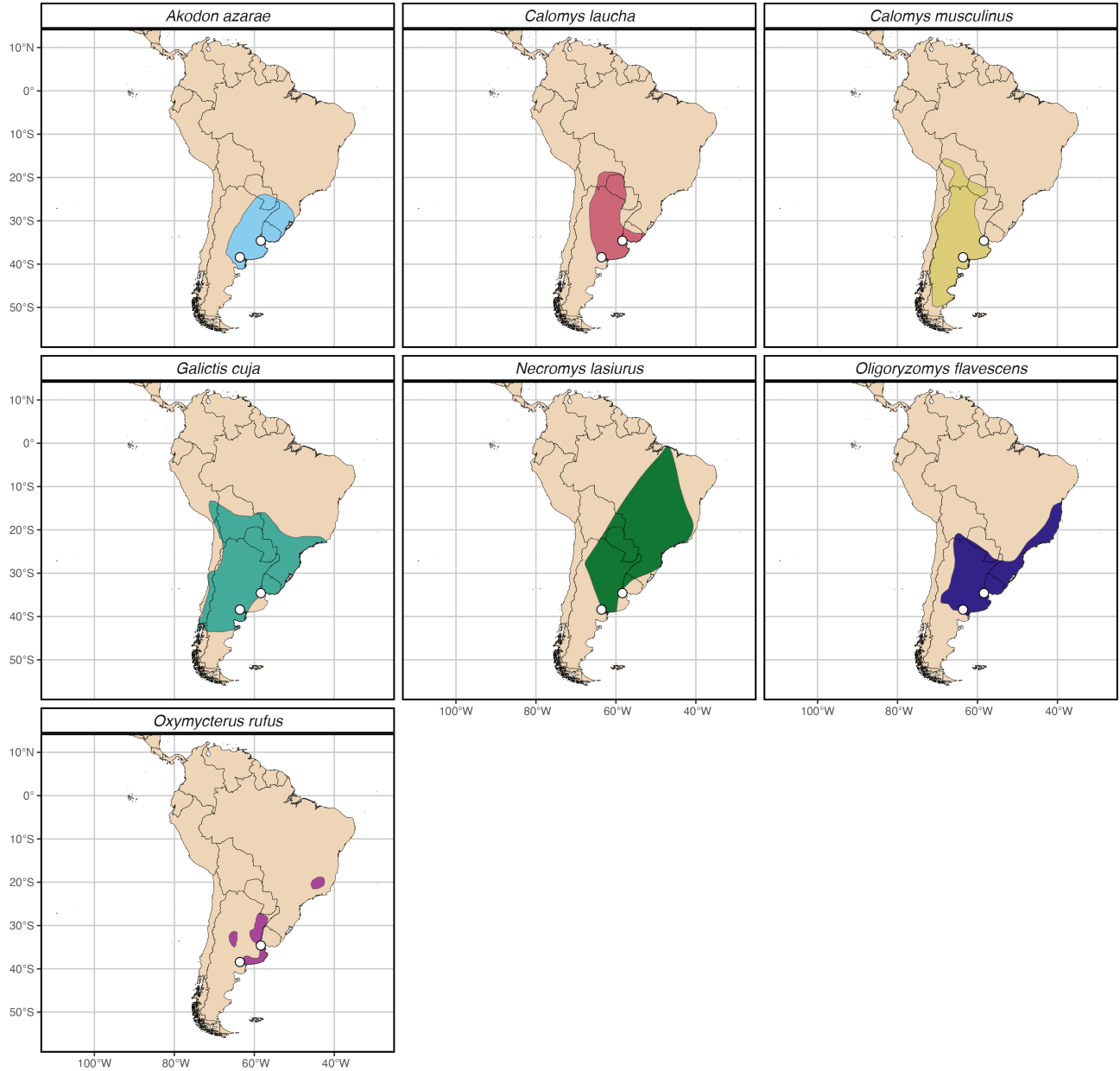

### Venezuelan Hemorrhagic Fever

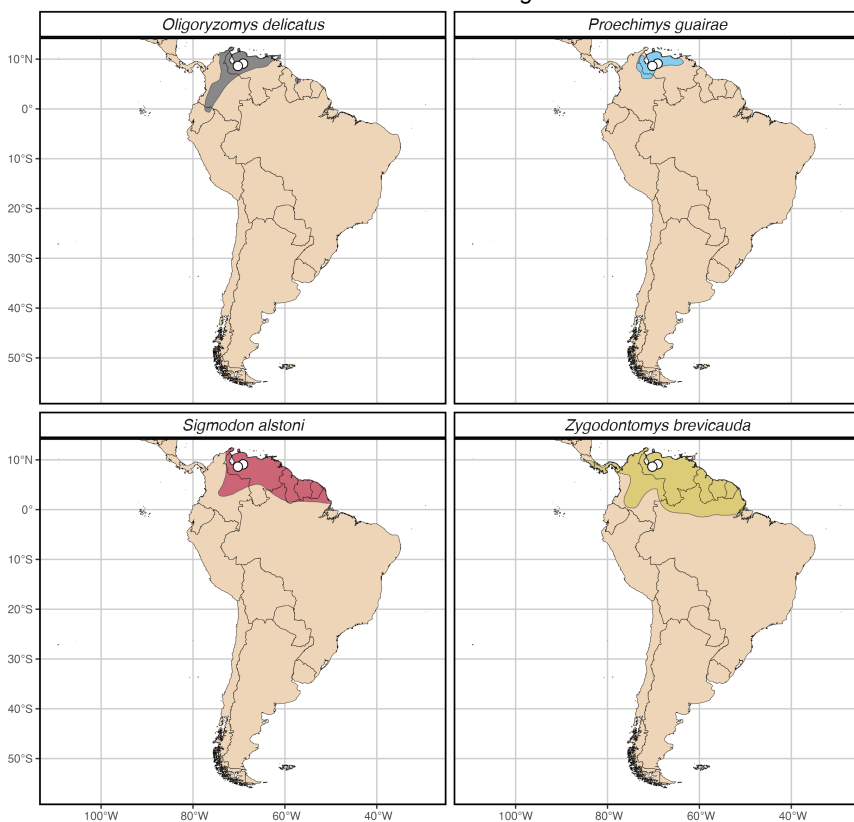

### Bolivian Hemorrhagic Fever

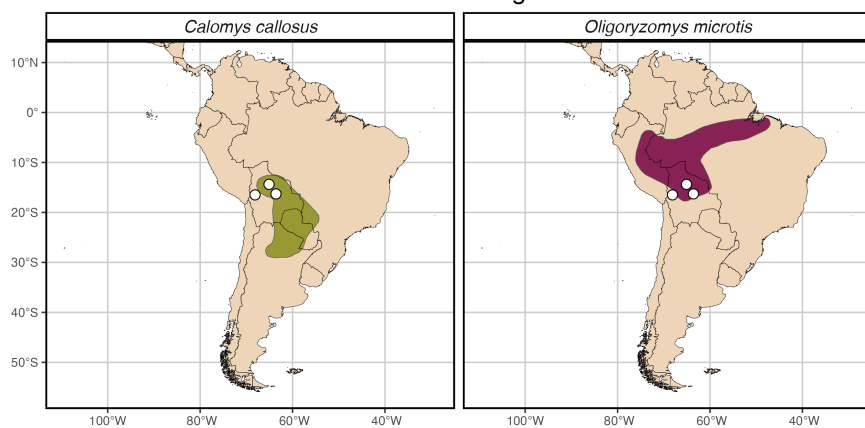

Supplement: Supplementary file 2 — Supplementary Material 2: Maps showing additional versions of Fig. 1 that include the distribution of each species alone and not overlapping [file 12866_2024_3257_MOESM2_ESM.pdf]
